# Breast Health Day 2013

**DOI:** 10.3332/ecancer.2013.ed28

**Published:** 2013-10-16

**Authors:** Giovanna Gatti

**Affiliations:** Senologist, scientific communication and new media, European Institute of Oncology, Milan, Italy

Held every year, Breast Health Day aims to disseminate information concerning breast health and raise awareness of prevention and early detection of breast cancer among women and girls across the globe.

Dr Giovanna Gatti, of the European Institute of Oncology, Milan, Italy, gives her own personal viewpoint on Breast Health Day 2013.

Breast Health Day is a day that should represent the whole year and the whole life for every woman. In principle, my work as a breast surgeon (mainly clinical: I prefer the relationship I can develop with women before and after surgery) at the European Institute of Oncology, should motivate me to speak about regular checks and correct examinations, but actually I think women do not need so much advice on carrying out examinations. They can obtain a large amount of information, from the media, on the internet and from their physicians any time they wish. Correct information should be an issue, but I do believe we are reaching a good level of widespread availability of correct information. There is an element of awareness that should be taken into account because quality of life and the compliance to physicians’ indications are as important as the technical issue of the secondary prevention of breast carcinoma. Women need to be understood, “loved” and followed during the entire development of consciousness and self-care responsibility because the decision to undertake breast cancer prevention is related to hope and happiness but also to fear, loneliness and feeling unsure.

“Make the right check at the right age and you will save your life”: in principle, this is a great message. For a certain section of the general population it is perfect advice to activate consciousness. For another section this is a statement of excessive responsibility and fear, and the result is not always participation in screening or campaigns. “Whenever I think that these exams could discover a small tumor I immediately run away”: the number of women scared of prevention is not minor. Moreover, we can consider this Breast Health Day to be an enormous tribute to all women who discovered their cancer and are now fighting, living a life with therapies and stress: for them the message should be different in terms of words but not less important or strong. Let’s hope, let’s be together to take into account all the needs and all the emotions involved.

I am very aware that cultures in different areas in Europe vary widely, and that a varied mix of belief, religion and accessibility to information strongly influences societies and – consequently – the likelihood of detecting very small breast lesions and being rapidly and efficiently cured in a high-level center. Our cultural level influences choice, and also our emotions play an important role. As a physician I hope to reach the maximum part of population with messages of prevention, reassurance and health. As a woman, I want to say that I understand. I feel and understand, and I send all my love to every woman reading here, now.

